# Draft Genome Sequence of *Acinetobacter baumannii* Strain ABBL099, a Multidrug-Resistant Clinical Outbreak Isolate with a Novel Multilocus Sequence Type

**DOI:** 10.1128/genomeA.00738-14

**Published:** 2014-09-11

**Authors:** Egon A. Ozer, Margaret A. Fitzpatrick, Alan R. Hauser

**Affiliations:** a Department of Medicine, Division of Infectious Diseases, Northwestern University, Chicago, Illinois, USA; b Department of Microbiology-Immunology, Northwestern University, Chicago, Illinois, USA

## Abstract

*Acinetobacter baumannii* is associated with hospital-acquired infections and can cause persistent outbreaks. Here we report the draft genome sequence of ABBL099, a multidrug-resistant clinical isolate of *A. baumannii* belonging to a novel sequence type and representative of clonal isolates cultured from patients at one institution over a 4-year time period.

## GENOME ANNOUNCEMENT

*Acinetobacter baumannii* is a common cause of hospital-acquired infections, particularly pneumonia and catheter-related bloodstream infections, and often causes clonal outbreaks in critically ill patients (1–3). *A. baumannii* infections have been associated with high attributable mortality and increased hospital length of stay (4). Multidrug resistance is often a predictor of poor clinical outcomes in patients infected with *A. baumannii* (5). *A. baumannii* is characterized by a significant capacity for long-term persistence in hospital environments, with clonal infections recurring in patients in the same hospital or unit over the course of years (6).

In this study, we determined the draft genome sequence of the *A. baumannii* multidrug-resistant strain ABBL099, a representative of a group of 11 clonal isolates collected between 2009 and 2012 from bloodstream infections in patients at Northwestern Memorial Hospital in Chicago, IL. Antibiotic susceptibilities were determined by Vitek2 (bioMérieux, France) (V), E tests (bioMérieux, France) (E), or disk diffusion (D). ABBL099 was found to be susceptible to ampicillin/sulbactam (V), tobramycin (V), and colistin (E) and resistant to imipenem (V), minocycline (D), doxycycline (D), amikacin (V), ciprofloxacin (V), and piperacillin/tazobactam (D). The tigecycline MIC was 8 mg/L (E). Whole-genome shotgun sequencing was performed on the Illumina HiSeq 2000 platform, generating 8,769,372 pairs of 101-bp paired-end reads with 250-bp inserts. Reads were randomly down sampled to yield approximately 150× genome coverage (2,970,296 read pairs) and *de novo* assembled using Ray v. 2.3.0 (7), generating 172 total contigs. The assembly was filtered for contigs larger than 200 bp, leaving 155 contigs with a total length of 3,917,723 bp, an *N*_50_ of 53,298 bp, and an average contig length of 25,276 bp. The overall G+C content was 38.9%.

Sequence annotation was performed using Prokka v. 1.9 (8), yielding 3,686 predicted protein-coding genes with an average gene length of 926 bp and a total length of 3,413,736 bp, covering 87.14% of the draft genome. In addition, 59 tRNA genes were identified and annotated, as well as 9 complete or partial rRNA gene annotations. ABBL099 was confirmed to belong to the species *A. baumannii* both through alignment of sequencing reads to the Silva 16S rRNA gene sequence database (9) as well as through evaluation of the *rpoB* gene sequence in the assembled contigs (10). The allele sequences of the *cpn60, fusA, gltA, pyrG, recA, rplB*, and *rpoB* genes were identified in the Institut Pasteur *A. baumannii* multilocus sequence typing (MLST) database (http://www.pasteur.fr/recherche/genopole/PF8/mlst/Abaumannii.html) and found to represent a previously unreported sequence type. We have since submitted the sequence type of ABBL099 to the Institut Pasteur database as ST 499.

### Nucleotide sequence accession numbers. 

This whole-genome shotgun project has been deposited at DDBJ/EMBL/GenBank under the accession number JPDG00000000. The version described in this paper is version JPDG01000000.
